# Evaluation of the pour-on administration of eprinomectin on milk yield and somatic cell counts in dairy ewes naturally infected with gastrointestinal nematodes

**DOI:** 10.1016/j.vpoa.2019.100016

**Published:** 2019-06-10

**Authors:** K. Arsenopoulos, A.I. Gelasakis, V. Delistamatis, E. Papadopoulos

**Affiliations:** a Laboratory of Parasitology and Parasitic Diseases, School of Veterinary Medicine, Faculty of Health Sciences, Aristotle University of Thessaloniki, 54124, Thessaloniki, Greece; b Laboratory of Farm Animal Anatomy and Physiology, Department of Animal Science and Aquaculture, School of Agricultural Production, Infrastructure and Environment, Agricultural University of Athens, Iera Odos, Athens, 11855, Greece; c Private Veterinary Practice, Soufli, Greece

**Keywords:** Eprinomectin, Dairy ewes, Efficacy, Nematodes, Milk yield, Somatic cell counts

## Abstract

The effect of pour-on eprinomectin administration on milk yield and somatic cell counts was studied in dairy ewes located on twelve farms in mainland Greece. On each farm, the selected ewes were randomly divided into three similar groups. Group 1 consisted of 10–15 untreated ewes (control group), Group 2 consisted of 10–13 ewes treated with a single dose of eprinomectin at Day 0 and Group 3 consisted of 6 ewes repeatedly treated with eprinomectin at Days 0, 42 and 70. Faecal egg counts and coprocultures were performed on Days 0, 7, 14, 28, 42, 56, 70, 84 and 98. The milk yield and somatic cell counts were measured. The topically administered eprinomectin was highly effective against gastrointestinal nematodes up to 42 days post treatment (94.1% and 99.7% for Groups 2 and 3, respectively). This beneficial effect was extended from Day 42 to 98, in ewes of Group 3. Ewes treated once or thrice presented an increase of daily milk yield by *ca.* 5% (50 mL/day) and 11% (105 mL/day), respectively, compared to untreated ewes. At the same frame, a significant decrease in somatic cell counts was observed in the eprinomectin treated ewes compared to the untreated ones. In conclusion, this study confirmed the high antiparasitic efficacy and the beneficial effect of pour-on eprinomectin on the milk yield and somatic cell counts in dairy ewes under semi-intensive management.

## Highlights

• Eprinomectin pour-on was highly effective against sheep nematodes.
• Eprinomectin pour-on increased daily milk yield of dairy ewes.
• Somatic cell counts were reduced improving the udder health status.

## 1 Introduction

Dairy sheep farming represents an important sector of livestock production in the Mediterranean basin, including Greece, with semi-intensive systems being the predominant ones. In these systems, feeding regimes are generally characterized by a combination of grazing on natural pasturelands and supplementary feeding of concentrates and roughages all year round (Zygoyiannis, 2006). Also, under semi-intensive systems, sheep are often heavily challenged by parasitic infections during grazing, which represent one of the most important causes of poor health and welfare and reduced productivity at flock level. In particular, parasitic infections may be associated with reduced milk and meat production, lamb growth and ewe fertility, increased culling and replacement rate and predisposition to other diseases, which explains the reason why they are considered a major issue for the sustainability of the farms (Papadopoulos et al., 2003, Laurenson et al., 2011, Geurden et al., 2014, Fthenakis et al., 2015, Mavrot et al., 2015, Hamel et al., 2017).

*Teladorsagia*, *Haemonchus*, *Trichostrongylus*, *Nematodirus* and *Chabertia* are the commonest nematode genera of the wide spectrum of gastrointestinal nematodes (GIN) infecting sheep, throughout Europe (Rehbein et al., 1999 ; Burgess et al., 2012; Domke et al., 2012; Hamel et al., 2017) and particularly among Mediterranean European countries, e.g. Spain (Uriarte et al., 2004), Italy (Cringoli et al., 2003, Torina et al., 2004) and Greece (Papadopoulos et al., 2003, Gallidis et al., 2009).

Routine anthelmintic treatments for the control of the fore mentioned parasites is the norm in sheep flocks, whereas, pasture management strategies are less commonly exploited (Silvestre and Humbert, 2002, Gelasakis et al., 2010, Kaplan and Vidyashankar, 2012). Although, evidence-based and targeted anthelmintic treatment remains the most effective strategy for the control of nematode infections (Sargison, 2011, Mavrot et al., 2015), the use of the commercially available anthelmintic drugs undergoes certain limitations, with the extended withdrawal periods during lactation and the development of resistance being the most significant ones (Hamel et al., 2017).

Macrocyclic lactones are among the most popular antiparasitic drugs. Eprinomectin is a modern macrocyclic lactone with a high efficacy against GIN, lungworms and some ectoparasites in cattle (Cringoli et al., 2003). A few years ago, to overcome the shortfall of anthelmintic drugs with zero withdrawal period in milk, the off-label use of eprinomectin was adopted by some dairy sheep farmers and it was not until recently that eprinomectin was registered for use in dairy sheep. Today, it represents a promising anthelmintic drug with easy and welfare-friendly administration (pour-on) and zero withdrawal period in milk in sheep (Imperiale et al., 2006).

The anthelmintic efficacy of pour-on eprinomectin has been studied in sheep (Hamel et al., 2017, Hamel et al., 2018), goats (Rehbein et al., 2014) and cattle (Rehbein et al., 2012). However, there is no research establishing the association between treatment of nematode-infected dairy sheep with eprinomectin and their milk yield or somatic cell counts (SCC) in milk. Therefore, the objective of the study was to evaluate the anthelmintic efficacy of eprinomectin and quantify its relationship with milk yield and SCC in milk of naturally infected dairy sheep flocks.

## 2 Materials and methods

### 2.1 Flocks history

Twelve flocks from three different regions (four farms per region) of the mainland Greece (Central Macedonia, Thessaly and Thrace) were included in the study. The average flock size was *ca.* 300 ewes with an average milk yield of about 290 L per ewe per lactation (250 days). The selected farms were representative of the typical semi-intensive dairy sheep farms in Greece, as described by Gelasakis et al. (2010). Their feeding regime was similar and included grazing on natural pastures for 6–8 h per day throughout the study and supplementary feeding with concentrates and alfalfa hay (up to 1.0 kg for each divided in 2 meals) and *ad libitum* access to wheat straw and water. All ewes were milked twice per day and were at the middle of lactation (5 months post-lambing).

All the flocks of the study were following similar vaccination and antiparasitic programs. Namely, the ewes were vaccinated against *Clostridium* spp. and *Pasteurella* spp. (Dialuene P^®^, MSD) 20 days before parturition and six months later. Another vaccination against *Mycoplasma agalactiae* (Agalax^®^, SYVA) was performed 30 days before parturition and repeated 6 months later. The antiparasitic program included the oral administration of albendazole (10 mg/kg BW), 30 days before lambing. Ewes involved in the experiment had not received any anthelmintic treatment in the past 4 months prior to the beginning of the study.

### 2.2 Experimental design

The study was conducted between April and August 2015. Three hundred and sixty clinically healthy adult (2nd–4th lactation) dairy ewes (26–36 ewes per farm) in good body condition score (between 2.5 and 3.5 in the five-grade scale proposed by Russel et al., 1969) were randomly selected and included in the study. All ewes were milked twice per day and were at the middle of lactation (5 months post-lambing).

On each farm, the selected ewes (from a pool of ewes expelling more than 300 epg) were randomly divided into three similar groups (Group 1, 2 and 3). Group 1 consisted of 10 to 15 untreated ewes (control group), Group 2 consisted of 10 to 13 ewes treated with a single dose of eprinomectin at Day 0 and Group 3 consisted of 6 ewes repeatedly treated with eprinomectin at Days 0, 42 and 70. The commercial product used for the study was Eprinex^®^ pour-on (Merial) which was applied directly onto the skin along the backline in a strip extending from the withers to the tail head, at the dose rate of 1.0 mg/kg BW per treatment.

### 2.3 Faecal sampling and parasitological procedures

Faecal samples were collected from each individual ewe of the three groups on Days 0, 7, 14, 28, 42, 56, 70, 84 and 98. These samples were collected directly from the rectum of the ewes, using single-use plastic gloves and lubricant. The samples were then stored and transferred to the laboratory in isothermal containers (2 to 4 °C). Sampling was done by a trained veterinarian and according to animal welfare principles and regulations to avoid any unnecessary distress for the animals.

Individual faecal egg counts (FEC) for nematode parasite eggs were assessed using a quantitative modified McMaster technique with a sensitivity of 50 eggs per g of faeces (EPG) (Ministry of Agriculture Fisheries and Food (MAFF), 1986).

Moreover, pooled faecal samples, from each farm, on the fore mentioned sampling occasions, were processed for coprocultures (Ministry of Agriculture Fisheries and Food (MAFF), 1986). The nematode larvae were recovered using the Baermann technique, after an incubation period of 7–10 days (Roberts and O’Sullivan, 1950). Morphological identification of 100 (when possible) parasitic nematode L_3_ larvae was performed according to morphological keys of Van Wyk and Mayhew (2013).

### 2.4 Milk sampling for the measurement of the Somatic Cell Counts

In every sampling occasion, individual milk samples were collected in the milking parlor from a subset of five selected ewes (the same every time) per group for each farm. Following proper disinfection of the udder with isopropyl alcohol and the withdrawal of the initial 2–3 squirts of milk, a milk sample (*ca*. 50 mL), was collected in plastic tube with lid, taking approximately equal volumes from each udder half. The measurement of SCC was performed using an automatic high-throughput analyzer Fossomatic^™^ FC (Foss).

### 2.5 Milk yield

During the sampling occasions, ewes were hand-milked and the volume of the produced milk per ewe per milking was recorded using a graduated measuring cylinder (Ilmenau Company). Daily milk yield (DMY) was estimated using one of the two milkings (morning or evening) and making the adjustments according to ICAR recommendations (AT4 method).

### 2.6 Statistical analysis

Data were analyzed using SPSS 23. Descriptives were calculated and followed by analytical statistics. Univariate analysis of variance was used to compare the average values of FEC, DMY and SCC between the three groups for each sampling occasion separately, after adjusting for the farm’s effect. The results were used to draw FEC, DMY and SCC curves for each of the three groups during the study. Three mixed linear regression models were built to quantify the effects of treatment with eprinomectin and the days post-treatment on FEC, DMY and SCC, for the g^th^ sampling occasion, of the hth ewe in the ith flock (EPG_ghi_) as described below:

EPG_ghi_ = μ + E_ghi_ + D_hi_ + *γ*_hi_ + *δ*_i_ + e_ghi_ (Model 1)

where: μ = intercept, E_ghi_ = fixed effect of treatment group (3 levels, 0 = no treatment, 1 = treatment with Eprinomectin at day 0, 2 = three treatments with Eprinomectin at days 0, 42 and 70), D_hi_ = fixed effect of sampling time (10 levels, 1st, 2nd,….. 10th sampling occasion), *γ*_hi_= repeated variation of the hth ewe in the ith flock, *δ*_i_ = random variation in the ith herd and e_ghi_ = residual error. First order autoregressive was used as covariance structure in the models.

For the models estimating the effects on DMY and SCC, the fixed effect of the regression coefficient of the parasitic load expressed in EPG was also calculated. In these models the levels of sampling time were 9. The assumptions of homoscedasticity, normal distribution and linearity for the models were checked by visually assessing the plots of standardized residuals against standardized predicted values and histograms, as well as the probability-probability and quantile-quantile plots of standardized residuals.

Species richness and species diversity of nematodes were calculated using the Menhinick’s (D) and Shannon (H) index, respectively, as described below:

$$D=\frac{S}{\sqrt{N}}$$

$$H=\sum_{i=1}^{S}p_{i}\;|\text{ln}p_{i}\text{| }$$

where S equals the number of different nematode species, N equals the total number of the sampled ewes and p_i_ represents the proportion of the total number of the parasites that are in species “i”. D-index was measured to demonstrate the number of different species of nematodes found in the studied sheep population. H-index was complementary used as a more reliable index of biodiversity accounting both for the numbers of species of nematodes and the dominance or evenness of species in relation to one another.

The overall efficacy of treatment with eprinomectin at each group was calculated as follows:

$$\left(\text{\%}\right)\text{ efficacy=100×}\frac{\text{C-T}}{\text{C}}$$

where C represents the arithmetic mean FEC for control ewes (Group 1) and T the arithmetic mean FEC for treated ewes (Group 2 and 3). Statistical significance was set at a = 0.05 level.

## 3 Results

The topical application of eprinomectin (Eprinex^®^, Merial) was well tolerated and no local or general adverse reactions were observed, across the present study.

Overall, the most prevalent nematode parasites found in all studied regions at Day 0 were *Teladorsagia* spp. (67%), followed by *Haemonchus* spp. (28%) and *Trichostrongylus* spp. (2%). Furthermore, *Haemonchus* spp. presented a higher infection rate in ewes from Thessaly than in those of Macedonia and Thrace (Table 1).

**Table 1:** Number and proportion (%) of L_3_ larvae (per parasite taxon), mean species richness (Menhinick’s index) and species diversity (Shannon index) per group, in the three studied regions, at Days 0, 14 and 98.

	Region (no. of examined ewes)	Tel	Hae	Tri	Chab	Bun	D	H	
Day 0		Group 1							
	Macedonia (n = 47)	309 (78.2)	75 (18.9)	9 (2.3)	1 (0.3)	1 (0.3)	0.73	0.63	
	Thessaly (n = 44)	216 (55.7)	157 (40.4)	10 (2.6)	3 (0.8)	2 (0.5)	0.75	0.85	
	Thrace (n = 49)	290 (73.1)	95 (24.0)	9 (2.3)	1 (0.3)	1 (0.3)	0.71	0.69	

		Group 2							
	Macedonia (n = 42)	308 (78.4)	80 (20.4)	4 (1.0)	0 (0.0)	1 (0.2)	0.61	0.57	
	Thessaly (n = 44)	208 (53.3)	165 (42.3)	12 (3.1)	2 (0.5)	3 (0.8)	0.75	0.87	
	Thrace (n = 45)	289 (73.2)	103 (26.1)	2 (0.5)	0 (0.0)	1 (0.2)	0.59	0.62	

		Group 3							
	Macedonia (n = 24)	298 (75.6)	85 (21.6)	7 (1.8)	2 (0.5)	2 (0.5)	1.02	0.67	
	Thessaly (n = 23)	205 (54.9)	153 (41.0)	13 (3.5)	1 (0.3)	1 (0.3)	1.04	0.85	
	Thrace (n = 24)	281 (71.1)	102 (25.8)	8 (2.1)	2 (0.5)	2 (0.5)	1.02	0.73	

Day 14		Group 1							
	Macedonia (n = 45)	306 (78.9)	73 (18.8)	7 (1.7)	1 (0.3)	1 (0.3)	0.75	0.61	
	Thessaly (n = 41)	208 (53.3)	162 (41.5)	19 (4.9)	1 (0.3)	0 (0.0)	0.63	0.87	
	Thrace (n = 49)	293 (74.6)	91 (23.1)	8 (2.0)	0 (0.0)	1 (0.3)	0.57	0.65	

		Group 2							
	Macedonia (n = 42)	58 (55.7)	40 (38.5)	4 (3.8)	1 (1.0)	1 (1.0)	0.77	0.91	
	Thessaly (n = 44)	49 (59.0)	31 (37.3)	3 (3.7)	0 (0.0)	0 (0.0)	0.45	0.8	
	Thrace (n = 44)	48 (57.1)	30 (35.7)	4 (4.8)	1 (1.2)	1 (1.2)	0.75	0.94	

		Group 3							
	Macedonia (n = 24)	25 (64.1)	13 (33.3)	1 (2.6)	0 (0.0)	0 (0.0)	0.61	0.75	
	Thessaly (n = 22)	0 (0.0)	0 (0.0)	0 (0.0)	0 (0.0)	0 (0.0)	0	0	
	Thrace (n = 23)	0 (0.0)	0 (0.0)	0 (0.0)	0 (0.0)	0 (0.0)	0	0	

Day 98		Group 1							
	Macedonia (n = 44)	297 (75.5)	90 (22.9)	5 (1.3)	0 (0.0)	1 (0.3)	0.6	0.62	
	Thessaly (n = 39)	206 (52.2)	179 (45.3)	7 (1.7)	2 (0.5)	1 (0.3)	0.8	0.81	
	Thrace (n = 49)	280 (71.4)	101 (25.7)	8 (2.0)	1 (0.3)	2 (0.6)	0.71	0.72	

		Group 2							
	Macedonia (n = 40)	93 (60.0)	56 (36.1)	6 (3.9)	0 (0.0)	0 (0.0)	0.47	0.8	
	Thessaly (n = 44)	163 (53.6)	125 (41.1)	15 (4.9)	0 (0.0)	1 (0.4)	0.6	0.87	
	Thrace (n = 44)	166 (66.4)	75 (30.0)	7 (2.8)	0 (0.0)	2 (0.8)	0.6	0.77	

		Group 3							
	Macedonia (n = 24)	42 (59.1)	27 (38.0)	2 (2.9)	0 (0.0)	0 (0.0)	0.61	0.78	
	Thessaly (n = 23)	132 (53.6)	101 (41.1)	10 (4.1)	1 (0.4)	2 (0.8)	1.04	0.89	
	Thrace (n = 24)	148 (65.7)	72 (32.1)	4 (1.8)	0 (0.0)	1 (0.4)	0.82	0.74	

Tel: *Teladorsagia* spp., Hae: *Haemonchus* spp., Tri: *Trichostrongylus* spp., Chab: *Chabertia* spp., Bun: *Bunostomum* spp. D: Menhinick’s species richness index, H: Shannon species diversity index.

Group 1: No eprinomectin treatment (control group), Group 2: Eprinomectin treatment at day 0, Group 3: Eprinomectin treatment at days 0, 42 and 70.

The overall efficacy and the efficacy of eprinomectin treatment on the most pathogenic GIN genera i.e. *Teladorsagia* and *Heamonchus*, on Days 7, 14, 28, 42, 56, 70, 84 and 98 for Groups 2 and 3, are presented in Table 2, Table 3, respectively. In Group 2, the overall efficacy was higher than 95% at Days 7, 14 and 28, whereas, in Group 3 the overall efficacy was higher than 99% at least for the first 70 days after the first treatment (Table 2). Moreover, the individual efficacies against *Teladorsagia* and *Haemonchus* spp. confirmed the above findings (Table 3).

**Table 2:** Mean (±SD) faecal egg counts per group of ewes and the anthelmintic efficacy (%) of Eprinex^®^ across the study.

	Group 1	Group 2		Group 3	
Day	Faecal egg count	Faecal egg count	Efficacy	Faecal egg count	Efficacy
7	821.87 (±253.02)	11.06 (±6.52)	98.7	0 (±0)	100.0
14	964.66 (±275.34)	16.32 (±11.53)	98.3	1.39 (±3.24)	99.9
28	1085.11 (±315.75)	40.65 (±17.82)	96.3	1.39 (±3.24)	99.8
42	1059.21 (±256.73)	65.22 (±32.57)	93.8	3.75 (±7.25)	99.7
56	1052.38 (±230.74)	113.87 (±52.05)	89.2	6.67 (±8.52)	99.4
70	935.25 (±148.32)	148.11 (±62.61)	84.2	1.39 (±3.24)	99.9
84	739.01 (±155.380)	194.24 (±118.89)	73.7	25.14 (±15.43)	96.6
98	485.57 (±56.17)	194.30 (±69.87)	60.0	18.21 (±14.79)	96.2

Group 1: No eprinomectin treatment (control group), Group 2: Eprinomectin treatment at day 0, Group 3: Eprinomectin treatment at days 0, 42 and 70.

**Table 3:** Mean calculated number of L_3_ larvae of the genera *Teladorsagia* and *Haemonchus* and the anthelmintic efficacy (%) of Eprinex^®^ per parasite genus per sampling occasion.

	Parasite genera	Group 1	Group 2		Group 3	
		L_3_ larvae	L_3_ larvae	Efficacy	L_3_ larvae	Efficacy
**Day 7**	Tel	600.94	7.30	98.79	0.00	100.00
	Hae	200.88	3.11	98.45	0.00	100.00
**Day 14**	Tel	670.44	9.30	98.61	0.88	99.87
	Hae	270.97	6.11	97.75	0.47	99.83
**Day 28**	Tel	770.44	29.30	96.20	0.88	99.89
	Hae	288.42	10.64	96.31	0.47	99.84
**Day 42**	Tel	720.99	40.30	94.41	2.01	99.72
	Hae	320.97	24.21	92.46	1.60	99.50
**Day 56**	Tel	723.04	71.60	90.10	4.00	99.45
	Hae	316.47	40.42	87.23	2.20	99.30
**Day 70**	Tel	661.03	83.60	87.35	0.98	99.85
	Hae	250.97	53.42	78.71	0.37	99.85
**Day 84**	Tel	550.94	107.60	80.47	15.00	97.28
	Hae	169.02	73.42	56.56	6.84	95.95
**Day 98**	Tel	322.22	115.65	64.11	10.82	96.64
	Hae	152.27	70.16	53.92	6.72	95.59

Tel: *Teladorsagia* spp., Hae: *Haemonchus* spp.

Group 1: No eprinomectin treatment (control group), Group 2: Eprinomectin treatment at day 0, Group 3: Eprinomectin treatment at days 0, 42 and 70.

Table 1 summarizes the number and proportion (%) of L_3_ larvae (per parasite taxon), mean species richness (Menhinick’s index, D index) and species diversity (Shannon index, H index) per group, in the three studied regions, at Days 0, 14 and 98. Menhinick’s index values observed in the studied regions varied from 0.59 to 1.04, 0.00 to 0.77, 0.47 to 1.04, whereas, Shannon index varied from 0.57 to 0.87, 0.00 to 0.94 and 0.62 to 0.89, for Days 0, 14 and 98, respectively (Table 1). The highest D and H indexes were observed in Thessaly in all sampling occasions and studied groups, with the exception of Day 14 (Table 1).

**Fig. 1:**
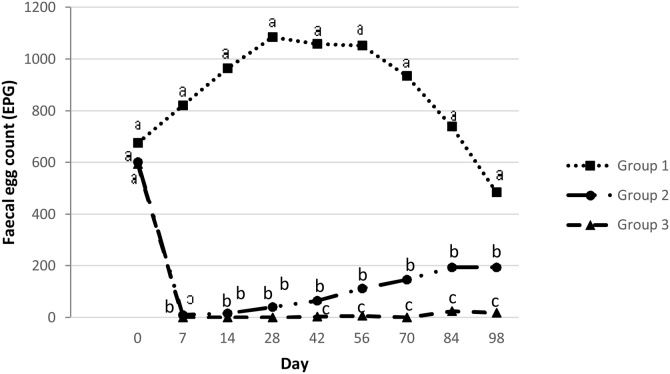
Mean curves, showing the effect of eprinomectin (Eprinex^®^, Merial) on faecal egg count (EPG) among three different groups of ewes per sampling occasion. a, b, c different superscripts indicate statistical differences (P<0.05) of faecal egg count among three groups of ewes per sampling. Group 1: No eprinomectin treatment (control group), Group 2: Eprinomectin treatment at day 0, Group 3: Eprinomectin treatment at days 0, 42 and 70.

As presented in Table 4, untreated ewes had significantly higher parasitic load when compared with ewes treated by eprinomectin once or thrice (increased by 522 and 606 EPG, respectively, P ≤ 0.001). The evolution of FEC (EPG) across the studied period for the three groups is presented in Fig. 1. FEC of Group 1 (untreated group) were significantly higher (P ≤ 0.05) than those of Group 2 and 3, in every sampling occasion after Day 0. Furthermore, FEC in Group 2 were significantly higher (P ≤ 0.05) than in Group 3 from Day 42 to Day 98.

**Table 4:** Effects of eprinomectin treatment and sampling on the faecal egg counts (EPG) in the studied ewes population.

Parameter	Category level	B	SE	P-value	Lower	Upper
					95% CI	
Treatment	No treatment	606	25.4	0.000	556	656
	Eprinomectin at 0 d	84	25.6	0.001	34	135
	Eprinomectin at 0, 42 and 70 d	“Ref”				
Sampling time	Day -14	232	21.7	0.000	189	274
	Day 0	328	20.8	0.000	288	369
	Day 7	35	20.5	0.091	−6	75
	Day 14	96	20.2	0.000	56	136
	Day 28	153	19.7	0.000	114	191
	Day 42	158	18.9	0.000	121	195
	Day 56	175	17.7	0.000	140	210
	Day 70	135	15.7	0.000	105	166
	Day 84	79	12.1	0.000	55	103
	Day 98	“Ref”				
Intercept	Continuous	22	23.8	0.363	−25	68

CI: Confidence interval; B: Coefficient; SE: Standard error; “Ref”: Reference category.

**Fig. 2:**
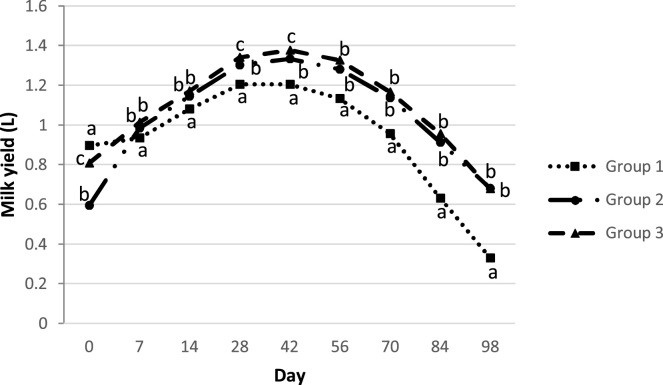
Mean curves showing the effect of eprinomectin (Eprinex^®^, Merial) on milk yield (L) among three different groups of ewes per sampling occasion. a, b, c different superscripts indicate statistical differences (P<0.05) of milk yield among three groups of ewes per sampling. Group 1: No eprinomectin treatment (control group), Group 2: Eprinomectin treatment at day 0, Group 3: Eprinomectin treatment at days 0, 42 and 70.

As presented in Table 5, in Groups 2 and 3, DMY was increased by *ca.* 5% (50 mL/day) and 11% (105 mL/day), respectively (P < 0.001), compared to Group 1 (untreated ewes Table 5). Fig. 2 demonstrates the milk yield curves of the three studied groups during the study period. Milk yield of the untreated ewes (Group 1) was significantly lower (P ≤ 0.05) when compared with milk yield of Groups 2 and 3, in every sampling occasion after Day 0. Furthermore, Group 3 produced significant (P ≤ 0.05) higher amount of milk than those of Group 2, on Days 28 and 42.

**Table 5:** Effects of eprinomectin treatment and sampling on the milk yield (L) in the studied ewes population.

Parameter	Category level	B	SE	P-value	Lower	Upper
					95% CI	
Treatment	No treatment	0.105	0.020	0.000	−0,144-0.144	−0.066
	Eprinomectin at 0 d	0.054	0.016	0.001	−0,085-0.085	−0.024
	Eprinomectin at 0, 42 and 70 d	“Ref”				
Sampling time	Day 0	0.231	0.018	0.000	0.195	0.268
	Day 7	0.421	0.017	0.000	0.387	0.456
	Day 14	0.578	0.017	0.000	0.544	0.612
	Day 28	0.729	0.017	0.000	0.695	0,764
	Day 42	0.752	0.017	0.000	0.718	0.785
	Day 56	0.691	0.017	0.000	0.658	0.724
	Day 70	0.530	0.016	0.000	0.500	0.561
	Day 84	0.274	0.013	0.000	0.248	0.299
	Day 98	“Ref”				
Intercept	Continuous	0.636	0.016	0.000	0.605	0.667

CI: Confidence interval; B: Coefficient; SE: Standard error; “Ref”: Reference category.

**Fig. 3:**
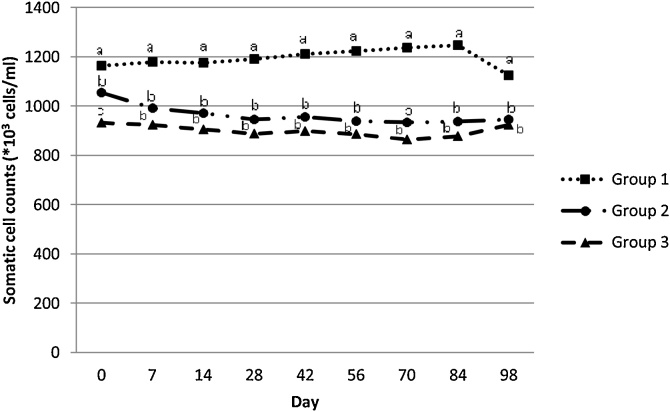
Mean curves, showing the effect of eprinomectin (Eprinex^®^, Merial) on somatic cell counts (*10^3^ cells/ml) among three different groups of ewes per sampling occasion. a, b, c different superscripts indicate statistical differences (P<0.05) of somatic cell counts among three groups of ewes per sampling. Group 1: No eprinomectin treatment (control group), Group 2: Eprinomectin treatment at day 0, Group 3: Eprinomectin treatment at days 0, 42 and 70.

Finally, as presented in Table 6, in Group 2 and 3 SCC were reduced by *ca.* 14% (165*10^3^ cells/mL) and 20% (229*10^3^ cells/ml), respectively, compared to those of Group 1. Fig. 3 presents the SCC curves of the three studied groups during the study period. SCC of the untreated ewes (Group 1) was significantly higher (P ≤ 0.05) when compared with SCC of Groups 2 and 3, in every sampling occasion after Day 0.

**Table 6:** Effects of eprinomectin treatment and sampling on the somatic cell counts (*10^3^ cells/ml) in the studied ewes population.

Parameter	Category level	B	SE	P-value	Lower	Upper
					95% CI	
Treatment	No treatment	229.386	38.483	0.000	153.361	305.411
	Eprinomectin at 0 d	64.551	37.675	0.089	−9.985	139.088
	Eprinomectin at 0, 42 and 70 d	“Ref”				
Sampling time	Day 0	32.969	24.369	0.177	−14.875	80.812
	Day 7	29.965	22.766	0.189	−14.729	74.658
	Day 14	14.636	21.930	0.505	−28.410	57.681
	Day 28	4.164	20.902	0.842	−36.859	45.188
	Day 42	18.123	19.456	0.352	−20.061	56.308
	Day 56	11.504	17.604	0.514	−23.045	46.053
	Day 70	9.650	15.039	0.521	−19.867	39.167
	Day 84	18.981	11.180	0.090	−2.963	40.926
	Day 98	“Ref”				
Intercept	Continuous	891.433	31.601	0.000	829.037	953.828

CI: Confidence interval; B: Coefficient; SE: Standard error; “Ref”: Reference category.

## 4 Discussion

This field trial assessed the beneficial effect of eprinomectin pour-on administration (once or thrice) on daily milk yield (DMY) and somatic cell counts (SCC) in twelve semi-intensive dairy sheep flocks, naturally infected with GIN.

The beneficial effect of the treatment with antiparasitic drugs (other than eprinomectin), such as albendazole, netobimin, moxidectin, on the DMY of dairy ewes has been previously confirmed in several studies (Jordan and Perez, 1991, Hoste and Chartier, 1993, Fthenakis et al., 2005). For example, Jordan and Perez (1991) recorded an increase of the DMY from ewes treated twice (4 weeks apart) with netobimin after lambing. In another study, the use of albendazole (3.8 mg/kg BW) and moxidectin (0.2 mg/kg BW) increased significantly the volume of the produced milk in dairy ewes of Lacaune breed post-weaning, under Greek conditions (Fthenakis et al., 2005). However, the above anthelmintic treatments are accompanied by limitations regarding their use during the lactation period due to the milk withdrawal period.

Eprinomectin is a macrocyclic lactone to be used as a topical formulation with high efficacy against nematodes of gastrointestinal track, respiratory system and some ectoparasites (Cringoli et al., 2003). This formulation is characterized by a broad safety margin and zero milk withdrawal period compared to other anthelmintic drugs (e.g. albendazole) due to a low milk partitioning coefficient, which is an unique pharmacokinetic property of the macrocyclic lactones class (Hamel et al., 2017). Even though, many surveys have confirmed its beneficial effect on DMY of grazing dairy cows, there is still lack of research regarding dairy ewes. Zaffaroni et al. (2000) reported that grazing dairy cows increased their DMY approximately 1.15, 1.46 and 5.52 L on days 20, 50 and 86 post-partum, respectively, after a single administration of pour-on eprinomectin, at the recommended dose rate, compared with the untreated control cows. At the same time, Reist et al. (2002) recorded a 2.14 L increase of daily milk production per cow within the first month post eprinomectin administration, when grazing period was over and the cows remained permanently housed. Similarly, Reist et al. (2011) confirmed the beneficial effect of topically administered eprinomectin on milk production of the cows due to reduction of nematode infection intensity, which has also been found to be the case in grazing dairy cows, treated during lactation or at calving (Gibb et al., 2005, Charlier et al., 2007). In contrast, there is one study in which no significant effect of eprinomectin treatment on milk production was observed; however, in the latter study the herds had no or limited grazing exposure (Sithole et al., 2005a).

Our results provide novel data to support the increase of DMY due to an anthelmintic administration, in particular the pour-on eprinomectin, in dairy ewes naturally infected by nematodes. An increase of 105 mL/day and 50 mL/day in DMY was observed for ewes treated thrice (Group 3) and once (Group 2), respectively, comparing to the control group (average DMY was *ca.* 970 mL). Hence, the total increase in milk yield was *ca.* 10.3 L and 4.9 L for ewes in Group 3 and 2, respectively, considering that the duration of the present study was 98 days. Previous studies conducted in dairy small ruminants have shown that GIN act by reducing the availability of nutrients that are likely to be partitioned for milk production (Jordan and Perez, 1991, Hoste and Chartier, 1993, Veneziano et al., 2004). Therefore, a possible explanation for the increase of milk production in our study is that a greater nutrient supply that was afforded by the treated ewes during the persistent period post eprinomectin administration was sufficient to enhance the milk production (Cringoli et al., 2008). It is obvious that further elucidation of these mechanisms should be done in order to maximize the benefit from the use of these anthelmintic protocols.

The increased milk yield resulted in about 9.27€ and 4.41€ extra profit per ewe for groups 3 and 2, respectively (0.90 €/L was the average milk price in Greece during the study). The cost per single administration of eprinomectin was estimated at 2.04 €/ewe (for a ewe weighing 60 kg, at a dose rate of 1 mg/kg BW). According to the calculations the total benefit for ewes treated thrice and ewes treated once was 3.25€ and 2.37€, whereas for the average sheep flock in the study (n = 300 ewes), the total benefit for the 98 days of the study could be 975€ and 711€, respectively. In any case, this is a rough estimation of the short term (mid to end of milking period) economic benefits from using eprinomectin. Longitudinal studies need to be undertaken to assess the direct and indirect benefits of eprinomectin (e.g. improvement of milk quality and ewes’ health and welfare status, better feed efficiency, reduced replacement rate, etc.) on farm’s economics and sustainability.

The reduction of the SCC in milk samples after the administration of the pour-on eprinomectin is a novel finding for dairy ewes, indicating an improvement of udder health status and milk quality post treatment. The beneficial effect of eprinomectin treatment on SCC has been studied in grazing cattle. For example, Reist et al. (2011) reported a reduction in SCC of grazing cows on the second day after the administration of pour-on eprinomectin on these animals. In agreement with the previous results, Zaffaroni et al. (2000) recorded a reduction in SCC in eprinomectin treated cows, when compared with untreated ones, on Days 20 and 50 post calving. It is well known that GIN parasitism and especially *Teladorsagia* spp. can lead to protein and therefore, immunoglobulin deficiency (Stear et al., 2003). GIN parasitism suppresses the defense mechanisms of the mammary gland of the ewe predisposing to increase of SCC and appearance of clinical (Mavrogianni et al., 2017) or subclinical mastitis (Kordalis et al., 2019). According to Coop and Kyriazakis (1999), reduced energy availability has been recorded during trichostrongylosis (i.e. parasites engage energy sources) which can lead to decreased neutrophil function (i.e. delayed mobilization to the mammary gland, impaired phagocytosis, inefficient intracellular killing) contributing to mastitis (Mavrogianni et al., 2017). In our study, eprinomectin administration reduced GIN burden in dairy ewes and lead to reduced SCC in milk by suppressing the forementioned mechanisms.

Based on the results of coprocultures, third stage larvae of *Teladorsagia*, *Haemonchus* and *Trichostrongylus* were most prevalent in the studied sheep population. It was evident that *Teladorsagia* remained the most commonly found nematode genus, even though an increase of *Haemonchus* infection has been recorded. These findings are in accordance with the results reported previously in large scale studies conducted in dairy sheep and goats in central and northern Greece (Papadopoulos et al., 2003, Gallidis et al., 2009).

A significant increase of FEC of untreated ewes (Group 1) was observed in our study. This sharp increase was more than double at the end of April (i.e. within a month from the start of the trial). The seasonal pattern for the increase of GIN parasite burdens in naturally infected ewes has been previously reported by Papadopoulos et al. (2003), who found that the nematode infection intensity started to increase significantly from February and peaked during spring. A possible explanation for this sharp increase is that the moisture-temperature levels favor parasite survival rates, which in combination with increased grazing time during spring, enhance the risk of infection (Papadopoulos et al., 2003, Jackson and Coop, 2007).

Efficacy studies, conducted in sheep (Hamel et al., 2017, Hamel et al., 2018), goats (Rehbein et al., 2014) and cattle (Rehbein et al., 2012), reported high anthelmintic efficacy of pour-on formulation of eprinomectin, at the recommended dosage, for each of the forementioned animal species. In our study, the efficacy of topically administered eprinomectin was also high (>90%) for the first 42 days post treatment. This beneficial effect was further extended for 60 days (i.e. from Day 42 to 98, in ewes treated thrice). In our study, a long term effect of eprinomectin was evidenced. This has also been reported by other studies assessing the effectiveness of pour-on eprinomectin against experimental or natural GIN infection of goats (Chartier et al., 1999) and cattle (Cramer et al., 2000) for a period over 28 days post treatment.

However, factors such as the fleece length (Cringoli et al., 2003), as well as the licking behavior (Laffont et al., 2003, Bousquet-Mélou et al., 2011), have been identified to interact with drug delivery and therefore, potentially with drug efficacy. For this reason, pour-on eprinomectin is recommended to be administered directly onto the skin from the withers to the start of tail after parting or shearing the fleece. It is important to note that the eprinomectin excipient limits the environmental accumulation of the drug, by reducing unabsorbed quantities to be rinsed off the fleece during a rainfall (Litskas et al., 2013). In cattle, rainfall, according to the SPC of the product, does not affect the absorbance of the drug when administered topically, but in sheep there is no relative data available (Eprinex, UK SPC, 2018). In order to overcome the possible limitations from pour-on administration in sheep, the oral administration of eprinomectin has been investigated and found to be highly effective as well (Badie et al., 2015). However, to the best of our knowledge, meat and milk residual analyses after the oral administration of eprinomectin in sheep are scarce.

## 5 Conclusion

This study confirmed the high efficacy of eprinomectin against the GIN of sheep. Ewes treated once or thrice had significantly lower FEC than the untreated ones. Furthermore, ewes treated thrice had lower FEC, than the ones treated once, from day 42 to day 98 (end of the study). It is reported for the first time the beneficial effect of pour-on eprinomectin on the daily milk yield of dairy ewes. Ewes treated once or thrice presented an increase of daily milk yield by *ca.* 5% (50 mL/day) and 11% (105 mL/day), respectively, compared to untreated ewes. Finally, the reduction of the somatic cell counts was an additional supporting result towards the improvement of udder health status and milk quality post eprinomectin treatment of dairy ewes.

## Declaration of Competing Interest

The authors declare that there is no conflict of interest.
